# Novel extracellular role of REIC/Dkk-3 protein in PD-L1 regulation in cancer cells

**DOI:** 10.1007/s00109-023-02292-w

**Published:** 2023-03-04

**Authors:** Yuma Gohara, Nahoko Tomonobu, Rie Kinoshita, Junichiro Futami, Léna Audebert, Youyi Chen, Ni Luh Gede Yoni Komalasari, Fan Jiang, Chikako Yoshizawa, Hitoshi Murata, Ken-ichi Yamamoto, Masami Watanabe, Hiromi Kumon, Masakiyo Sakaguchi

**Affiliations:** 1 Department of Cell Biology, Dentistry and Pharmaceutical Sciences, Okayama University Graduate School of Medicine, 2-5-1 Shikata-Cho, Kita-Ku, Okayama-Shi, Okayama, 700-8558 Japan; 2 Department of Interdisciplinary Science and Engineering in Health Systems, Okayama University, Okayama, Japan; 3 Department of Urology, Dentistry and Pharmaceutical Sciences, Okayama University Graduate School of Medicine, Okayama, Japan; 4 Innovation Center Okayama for Nanobio-Targeted Therapy, Okayama University, Okayama, Japan; 5 Sorbonne Université, Collège Doctoral, Paris, 75005 France; 6 Department of General Surgery & Bio-Bank of General Surgery, The Fourth Affiliated Hospital of Harbin Medical University, Harbin, 150001 China; 7 Faculty of Medicine, Udayana University, Denpasar, Bali, Indonesia

**Keywords:** Breast cancer, REIC/Dkk-3, PD-L1, Immune checkpoint, Cancer therapy

## Abstract

**Abstract:**

The adenovirus-REIC/Dkk-3 expression vector (Ad-REIC) has been the focus of numerous clinical studies due to its potential for the quenching of cancers. The cancer-suppressing mechanisms of the *REIC/DKK-*3 gene depend on multiple pathways that exert both direct and indirect effects on cancers. The direct effect is triggered by REIC/Dkk-3-mediated ER stress that causes cancer-selective apoptosis, and the indirect effect can be classified in two ways: (i) induction, by Ad-REIC-mis-infected cancer-associated fibroblasts, of the production of IL-7, an important activator of T cells and NK cells, and (ii) promotion, by the secretory REIC/Dkk-3 protein, of dendritic cell polarization from monocytes. These unique features allow Ad-REIC to exert effective and selective cancer-preventative effects in the manner of an anticancer vaccine. However, the question of how the REIC/Dkk-3 protein leverages anticancer immunity has remained to be answered. We herein report a novel function of the extracellular REIC/Dkk-3—namely, regulation of an immune checkpoint via modulation of PD-L1 on the cancer-cell surface. First, we identified novel interactions of REIC/Dkk-3 with the membrane proteins C5aR, CXCR2, CXCR6, and CMTM6. These proteins all functioned to stabilize PD-L1 on the cell surface. Due to the dominant expression of CMTM6 among the proteins in cancer cells, we next focused on CMTM6 and observed that REIC/Dkk-3 competed with CMTM6 for PD-L1, thereby liberating PD-L1 from its complexation with CMTM6. The released PD-L1 immediately underwent endocytosis-mediated degradation. These results will enhance our understanding of not only the physiological nature of the extracellular REIC/Dkk-3 protein but also the Ad-REIC-mediated anticancer effects.

**Key messages:**

• REIC/Dkk-3 protein effectively suppresses breast cancer progression through an acceleration of PD-L1 degradation.

• PD-L1 stability on the cancer cell membrane is kept high by binding with mainly CMTM6.

• Competitive binding of REIC/Dkk-3 protein with CMTM6 liberates PD-L1, leading to PD-L1 degradation.

**Supplementary Information:**

The online version contains supplementary material available at 10.1007/s00109-023-02292-w.

## Introduction

The novel tumor suppressor gene *REIC* (*reduced expression in immortalized cells*) was first identified by Dr. Toshiya Tsuji in 2000 under the supervision of Dr. Masayoshi Namba [1]. Because *REIC* and *DKK3* (*Dickkopf WNT signaling pathway inhibitor 3*) are the same gene, they are referred to as the *REIC/DKK3* gene [1]. We have been investigating the protein nature of REIC/Dkk-3 and its usefulness in clinical applications for cancer patients. Our basic research has unveiled several aspects of the unique nature of the REIC/Dkk-3 protein [2, 3]. When REIC/Dkk-3 is overexpressed with the use of a gene-engineered adenovirus-REIC/Dkk-3 expression vector (hereinafter Ad-REIC), REIC/Dkk-3 induces apoptotic cell death in cancer cells while sparing normal cells [4–13]. This apoptotic event is triggered by a form of endoplasmic reticulum (ER) stress that stems from the compiled foreign REIC/Dkk-3 protein within the ER in the infected cells, which then activates a c-Jun N-terminal kinase/stress-activated protein kinase (JNK/SAPK)–Bax pathway [4].

The expression vector Ad-REIC also induces a form of ER stress in normal cells after the infection, but notably the normal cells are not killed, possibly indicating that normal cells have a different sensitivity to this particular form of ER stress compared to cancer cells. In fact, in Ad-REIC-infected normal cells, Ad-REIC-induced ER stress activates p38 but not JNK/SAPK, with p38, in turn, activating the signal transducers and activators of the transcription 1 (STAT1)-interferon regulatory factor 1 (IRF1) pathway, which finally leads to the induction of interleukin 7 (IL-7) [14]. The induction of a high level of IL-7 has been shown to indirectly contribute to cancer cell death in vivo by way of activation of natural killer (NK) cells in nude mice [14]. Due to the exciting potential of the *REIC/DKK3* gene and the vector Ad-REIC for anticancer therapy, investigations of Ad-REIC have progressed to clinical studies of patients with prostate cancer, liver cancer, and recurrent malignant glioma [15–19].

However, the above-described events may not constitute an intrinsic physiological role of the REIC/Dkk-3 protein, in light of the extremely high-level expression of foreign REIC/Dkk-3 protein in cells. Its nature as a secretory protein suggests that REIC/Dkk-3 also plays an intrinsic extracellular role. Bearing this in mind, we previously investigated the extracellular role of REIC/Dkk-3 and revealed that REIC/Dkk-3 plays a role in the differentiation of monocytes to dendritic-like cells, with the resulting dendritic-like cells contributing to tumor regression through their activation of cytotoxic T cells in vivo [2, 3]. Bhaloo et al. described their interesting findings that (i) C-X-C chemokine receptor type 7 (CXCR7) serves as a prominent REIC/Dkk-3 receptor and (ii) the binding of REIC/Dkk-3 to CXCR7 stimulates the migration of vascular progeny cells [20]. We speculated that, based on the functional similarity among the members of the chemokine receptor family, REIC/Dkk-3 may have one or more similar receptors. We conducted the present study to clarify the anticancer role of the REIC/Dkk-3-receptor(s) axis, the precise knowledge of which would contribute to our understanding of the physiological roles of the REIC/Dkk-3 protein and would also provide further evidence that Ad-REIC mediates cancer effects.

## Materials and methods

### Cells

HEK293T cells, a non-cancerous human embryonic kidney epithelial cell line with a stable expression of the SV40 large T antigen, were obtained from the RIKEN BioResource Center (Tsukuba, Japan). Human pancreatic cancer PANC-1, AsPC-1, and MIA PaCa-2 cells, human breast cancer MDA-MB-231 and MCF-7 cells, human prostate cancer PC-3 and LNCaP cells, human squamous cancer A431 cells, human cervical cancer HeLa cells, human melanoma MeWo cells, human mesothelioma MSTO-211H cells, and human lung cancer NCI-H2170 cells were obtained from the American Type Culture Collection (Rockville, MD). All cell lines were cultivated in DMEM/F12 (D/F) medium (Thermo Fisher Scientific, Waltham, MA) supplemented with 10% fetal bovine serum (FBS).

Human neutrophils were isolated from the blood of a healthy donor by the conventional method. In brief, the polymorphonuclear cell fraction was first collected from the whole blood by centrifugation at 2000 rpm for 30 min using the PolymorphPrep™ solution (Serumwerk Bernburg, Bernburg, Germany). The collected cells were then incubated with CD16 microbeads (Miltenyi Biotec, Bergisch Gladbach, Germany), and the unbound flow through the fraction was collected as the neutrophil-enriched fraction.

### Expression plasmids

To express the human genes of interest at high levels, we used our original pIDT-SMART (C-TSC) vector, the short name of which is the pCMViR-TSC vector [21]. All of the constructs were designed to be expressed in a C-terminal 3Flag-6His-tagged, 3Myc-6His-tagged, or 3HA-6His-tagged form. Transient transfection of these plasmids into cultured cells was performed using the transfection reagent FuGENE-HD^®^ (Promega BioSciences, San Luis Obispo, CA).

### Recombinant REIC/Dkk-3 protein

High-purity human recombinant REIC/Dkk-3 protein was prepared as described previously [3].

### Quantitative RT-PCR

ISOGEN II Isolation Reagent was used to extract total RNA from all cell types prepared in this study (Nippon Gene, Tokyo). Reverse transcription was then performed using ReverTraAce qPCR RT Master Mix with gDNA Remover (Toyobo, Osaka, Japan). Real-time polymerase chain reaction (PCR) was performed using FastStart SYBR^®^ Green Master Mix (Roche Applied Science, Penzberg, Germany) with specific primers on a StepOnePlus™ Real-time PCR system (Applied Biosystems, Foster, CA).

The primers used were as follows: *ACTB* (forward: 5′-ctggaacggtgaaggtgaca-3′; reverse: 5′-aagggacttcctgtaacaatgca-3′); *REIC/DKK3* (forward: 5′-aactgatggaggacacgcag-3′; reverse: 5′-gctgggaggtaagtttgcca-3′); *CD274* (*PD-L1)* (forward: 5′-ggcatccaagatacaaactcaa-3′; reverse: 5′-cagaagttccaatgctggatta-3′); *C5AR* (forward: 5′-ggagggaccttcgatcctc-3′; reverse: 5′-ggggtggtataattgaaggagtt-3′); *CXCR2* (forward: 5′-gaggcacagtgaagacatcg-3′; reverse: 5′-gctgggcttttcacctgtag-3′); *CXCR6* (forward: 5′-ggggatgacatgtgactcctat-3′; reverse: 5′-cgtgctcacctcttcaacct-3′); *ACKR3* (*CXCR7)* (forward: 5′-cagttgttgcaaagtgctcag-3′; reverse: 5′-cgggcaatcaaatgacct-3′); and *CMTM6* (forward: 5′-ggacttcagctgagattgctg-3′; reverse: 5′-ccctagtggtattttcaggttttc-3′).

### Immunoprecipitation (IP)

Monoclonal anti-DYKDDDDK tag agarose (clone 1E6; Fujifilm Wako Pure Chemical, Osaka, Japan) that recognizes Flag tag, monoclonal anti-HA tag agarose (Sigma-Aldrich, St. Louis, MO), or monoclonal anti-Myc tag agarose (MBL, Nagoya, Japan) was used for the immunoprecipitation experiments to capture the ectopically overexpressed proteins. The tag-agarose beads were mixed with various cell extracts and incubated for 3 h at 4 °C. After incubations of the individual samples, the bound proteins were pulled down by centrifugation. The precipitated proteins were subjected to sodium dodecyl sulfate–polyacrylamide gel electrophoresis (SDS-PAGE).

### Western blotting (WB)

Western blotting was done according to a standard method. The antibodies used were as follows: mouse anti-HA tag antibody (clone 6E2; Cell Signaling Technology [CST], Danvers, MA), mouse anti-Myc tag antibody (clone 9B11; CST), mouse anti-Flag tag antibody (clone M2; Sigma-Aldrich), rabbit anti-human phospho-p44/42 MAPK (Erk1/2) (Thr202/Tyr204), rabbit anti-human p44/42 MAPK (Erk1/2), rabbit anti-human phospho-SAPK/JNK (Thr183/Tyr185), rabbit anti-human SAPK/JNK (CST), rabbit anti-human phospho-Akt (Ser473), rabbit anti-human Akt, mouse anti-human C5aR antibody (Bio-Rad Laboratories, Hercules, CA), rabbit anti-human CXCR2 antibody (Thermo Fisher Scientific), rabbit anti-human CXCR6 antibody (GeneTex, Irvine, CA), rabbit anti-human CXCR7 antibody (Proteintech, Rosemon, IL), mouse anti-human CMTM6 antibody (used as ab1) (Absea Biotechnology, China), rabbit anti-human CMTM6 antibody (used as ab2) (Sigma-Aldrich), mouse anti-GFP antibody (Thermo Fisher Scientific), rabbit anti-GAPDH (CST), mouse anti-α-tubulin (Sigma-Aldrich), and mouse anti-β-actin (Sigma-Aldrich) antibodies.

### Protein identification

The collected cut-out piece from the silver-stained gel was trypsinized in a trypsin digestion buffer (10 mM CaCl_2_, 100 mM ammonium bicarbonate, pH 7.8) overnight at 37 °C. The digested sample was subjected to protein identification using a nano-flow liquid chromatography-mass spectrometry apparatus (Agilent 6330 Ion Trap; Agilent Technologies, Santa Clara, CA) equipped with an analytical chip (Agilent HPLC-Chip; Agilent Technologies). The resulting tandem mass spectrometry spectra of the tryptic peptides were analyzed using Agilent software (Spectrum Mill MS Proteomics Workbench; Agilent Technologies) with the protein database (SwissProt) for putative Homo sapiens protein identifications.

### NK cell-based in vitro assay

After being mixed with PolymorphPrep, blood from a healthy donor was centrifuged at 2000 rpm for 30 min, and the mononuclear cell fraction was collected. NK cells were then isolated from the collected fraction with the use of an NK cell isolation kit (Miltenyi Biotec) according to the manufacturer’s instructions. The interferon-gamma (IFN-γ) production and cancer cell cytotoxicity activities of the isolated NK cells were evaluated in vitro. For the assessment of IFN-γ induction, the isolated NK cells were stimulated with IL-2, an enhancer of NK cell proliferation and cytotoxicity (PeproTech, Rocky Hill, NJ) at 12 ng/ml for 72 h. The matured NK cells (2 × 10^4^ cells) were then mixed with or without MDA-MB-231 cells (1 × 10^4^ cells) in the presence of REIC/Dkk-3 recombinant protein or control bovine serum albumin (BSA) at 50 μg/ml and cultured for another 24 h.

After the cultivations, the IFN-γ levels in the collected supernatants were measured by an enzyme-linked immunosorbent assay (ELISA) (Human IFN-γ DuoSet ELISA; R&D Systems, Minneapolis, MN). In the assessment of the induction of IFN-γ, NK-cell-mediated cancer cell cytotoxicity was also evaluated. Matured NK cells (5 × 10^4^ cells) prepared by treatment with IL-2 (12 ng/ml, 72 h) were mixed with cell-permeant dye Calcein-AM (10 μM) (Thermo Fisher Scientific)-labeled MDA-MB-231 cells (1 × 10^4^ cells) that were treated with either REIC/Dkk-3 or BSA at the concentration of 50 μg/ml for 4 h. After another 4-h cultivation of the mixed cells in the presence of the same concentration of REIC/Dkk-3 or BSA, the cleared supernatants were collected and the fluorescence of the released Calcein-AM from the injured MDA-MB-231 cells was measured.

### Animal experiments

This animal study was approved by the Animal Care and Use Committee of Okayama University (approval no. OKU-2021446). All mouse procedures and euthanasia were done on anesthetized mice, strictly following the committee’s guidelines. MDA-MB-231 cells (5 × 10^6^ cells) mixed with Matrigel^®^ matrix (Corning, Corning, NY) in a 1:1 volume ratio were subcutaneously transplanted into BALB/c-nu/nu mice (Charles River Laboratories Japan, Yokohama, Japan). Six mice were examined in each group. The sizes of the tumors were measured with a vernier caliper, and tumor volume was calculated as 1/2 × (shortest dia.)^2^ × (longest dia.). Paraffin-embedded specimens of the excised tumors in all animal experiments were histologically examined by immunohistochemistry.

### Immunocytochemistry (ICC)

For the visualizations of programmed cell death 1 (PD-1) ligand 1 (PD-L1) and F-actin, cultured MDA-MB-231 cells were fixed with 4% paraformaldehyde (PFA) and washed with 0.1% Triton X-100 in phosphate-buffered saline (PBS). PD-L1 was stained with rabbit anti-human PD-L1 antibody (GeneTex, Irvine, CA) as the primary antibody and further treated with Alexa594-conjugated goat anti-rabbit IgG antibody (Thermo Fisher Scientific) as the secondary antibody. For the staining of actin filaments (F-actin), Alexa488-conjugated phalloidin (Thermo Fisher Scientific) was used.

### Immunohistochemistry (IHC)

Paraffin-embedded tumor tissues resected from mice were deparaffinized, and antigen retrieval was performed by conventional microwave treatment using a citric acid solution. Endogenous peroxide activity was quenched by treatment of the specimens with 3% hydrogen peroxide (Kanto Chemical Co., Hokkaido, Japan) for 10 min at room temperature (RT). The specimens were then incubated overnight at 4 °C with rabbit anti-human PD-L1 antibody (GeneTex). After being washed with 0.05% Tween-20 in PBS, the slides were incubated with horseradish peroxidase (HRP)-labeled secondary antibody. Signals were visualized by treating the antibody-reacted slides with a substrate, 3,3′-diaminobenzidine (*DAB*) (DAB Substrate Kit; Vector, Burlingame, CA). Apoptotic cells were visualized by terminal deoxynucleotidyl transferase (TdT)-mediated dUTP nick-end labeling (TUNEL) staining.

### Statistical analysis

Data are expressed as the mean ± SD. We used a simple pair-wise comparison with Student’s *t*-test (two-tailed distribution with two-sample equal variance). Probability (*p*)-values < 0.05 were considered significant.

## Results

### Finding of REIC/Dkk-3 protein-interacting membrane protein(s)

**Fig. 1:**
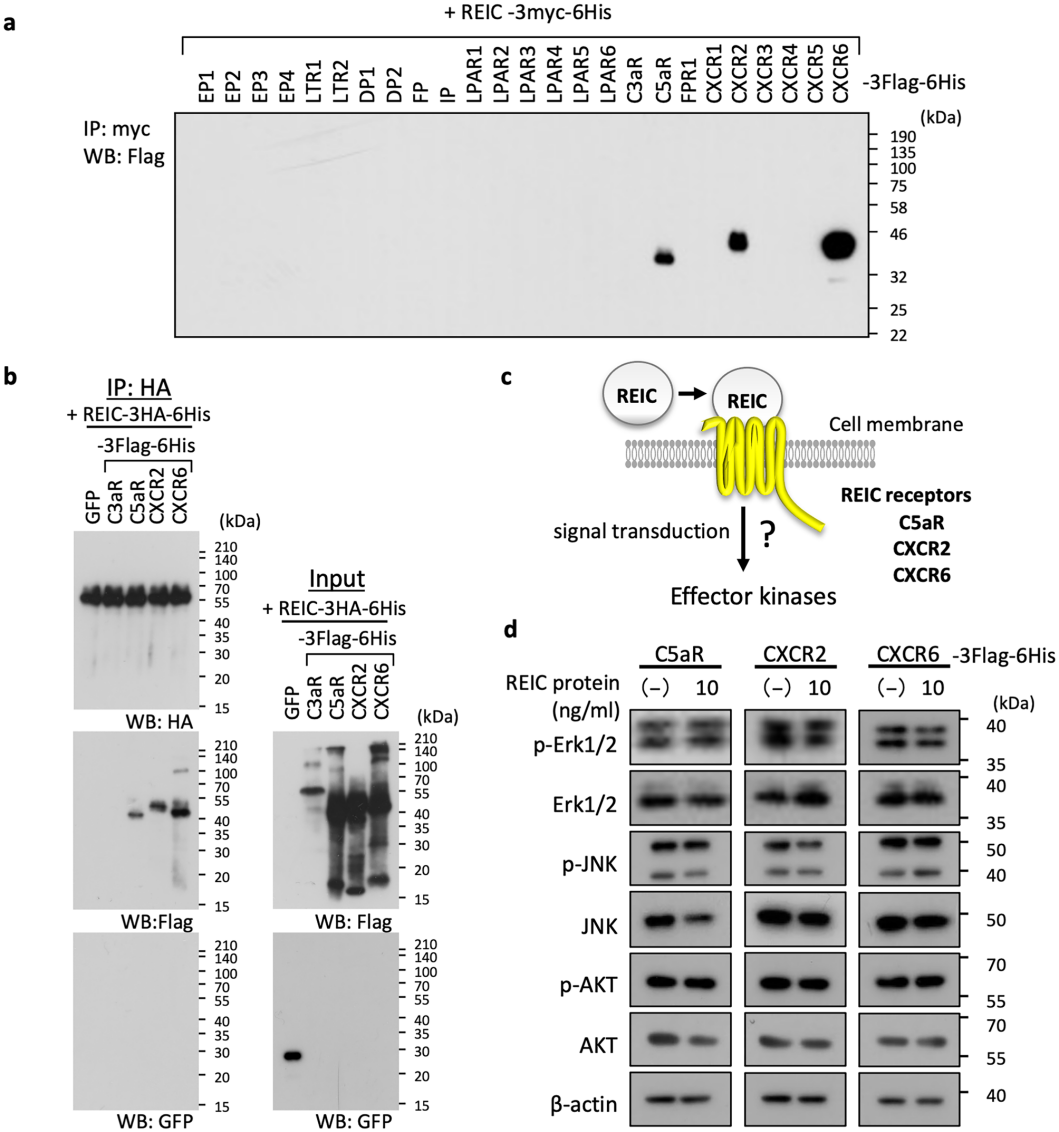
Candidate-based screening of the REIC/Dkk-3 interaction. **a** HEK293T cells were co-transfected with the following Myc-tagged REIC/Dkk-3 and Flag-tagged chemokine receptors: EP1-3 (prostaglandin E receptor 1–3: gene name, *PTGER1-3*), LTR1-2 (cysteinyl leukotriene receptor 1, 2: gene names, *CYSLTR1, 2*), DP1, 2 (prostaglandin D2 receptor: gene names, *PTGDR, PTGDR2*), FP (prostaglandin F receptor: gene name, *PTGFR*), IP (prostaglandin I2 receptor: gene name, *PTGIR*), LPAR1-6 (lysophosphatidic acid receptor 1–6: gene name, *LPAR1-6*), C3aR (complement C3a receptor 1: gene name, *C3AR1*), C5aR (complement C5a receptor 1: gene name, *C5AR*), FPR1 (formyl peptide receptor 1: gene name, *FPR1*), and CXCR1-6 (C-X-C motif chemokine receptor 1–6: gene name, *CXCR1-6*). After the immunoprecipitation of the expressed REIC/Dkk-3 with Myc antibody-conjugated beads, interacting receptors were detected by the Flag antibody. **b** Co-transfection of REIC/Dkk-3-3HA-6His with individual membrane proteins (C3aR, C5aR, CXCR2, CXCR6) tagged with 3Flag-6His or GFP was performed in HEK293T cells. 24 h after the transfections, cell extracts were prepared and then immunoprecipitated using anti-HA tag beads for the expressed REIC/Dkk-3, and a 4-μl volume of each specimen was analyzed by western blotting. **c** The binding of REIC/Dkk-3 to the identified REIC receptor candidates was expected to induce activation of some signal transduction pathways that lead to phosphorylation enhancements of several effector kinases. **d** HEK293T cells were transiently transfected with the identified candidate receptors for 24 h and then treated or not treated with REIC/Dkk-3 (10 ng/ml) for 1 h. The treated cells were lysed and subjected to western blotting with the indicated antibodies

Because the extracellular REIC/Dkk-3 protein has been shown to interact with the chemokine receptor CXCR7 [20], we first investigated potential interaction(s) of REIC/Dkk-3 with other chemokine receptors, including CXCR2, an important paralog of CXCR7, since chemokine receptors often share the same ligands. Our previously described screening method [22] identified positive signals of the interaction (Fig. 1a). The candidates were C5aR, CXCR2, and CXCR6. That these chemokine receptors underwent the expected interactions was further confirmed by another IP-WB method similar to that described in the legend of Fig. 1a; this method uses two different negative controls, GFP and C3aR, whose binding with REIC/DKK-3 was not detected in the screening experiment, as shown in Fig. 1a, b. We then stimulated the candidate receptors with the recombinant REIC/Dkk-3 protein and studied the changes in signal transduction, focusing on the effector kinases, i.e., ERK1/2 and AKT, in the force-expressed cells of the candidate receptors (Fig. 1c) because these kinases are activated with their phosphorylation modification beneath CXCR7 upon REIC/Dkk-3 binding [20].

However, contrary to our expectations, none of the candidates induced any appreciable change in phosphorylation for ERK1/2 and AKT (even with additional kinase JNK for this analysis) after stimulation with REIC/Dkk-3 at 10 ng/ml, an REIC/Dkk-3 concentration within the range used elsewhere [20] (Fig. 1d). This result revealed another potential action of REIC/Dkk-3 on these receptors independent of mere signal transduction.

### The REIC/Dkk-3 interactive membrane protein(s) binds with PD-L1 and the bound complexes lead to the stable presence of PD-L1 on the cancer cell membrane

**Fig. 2:**
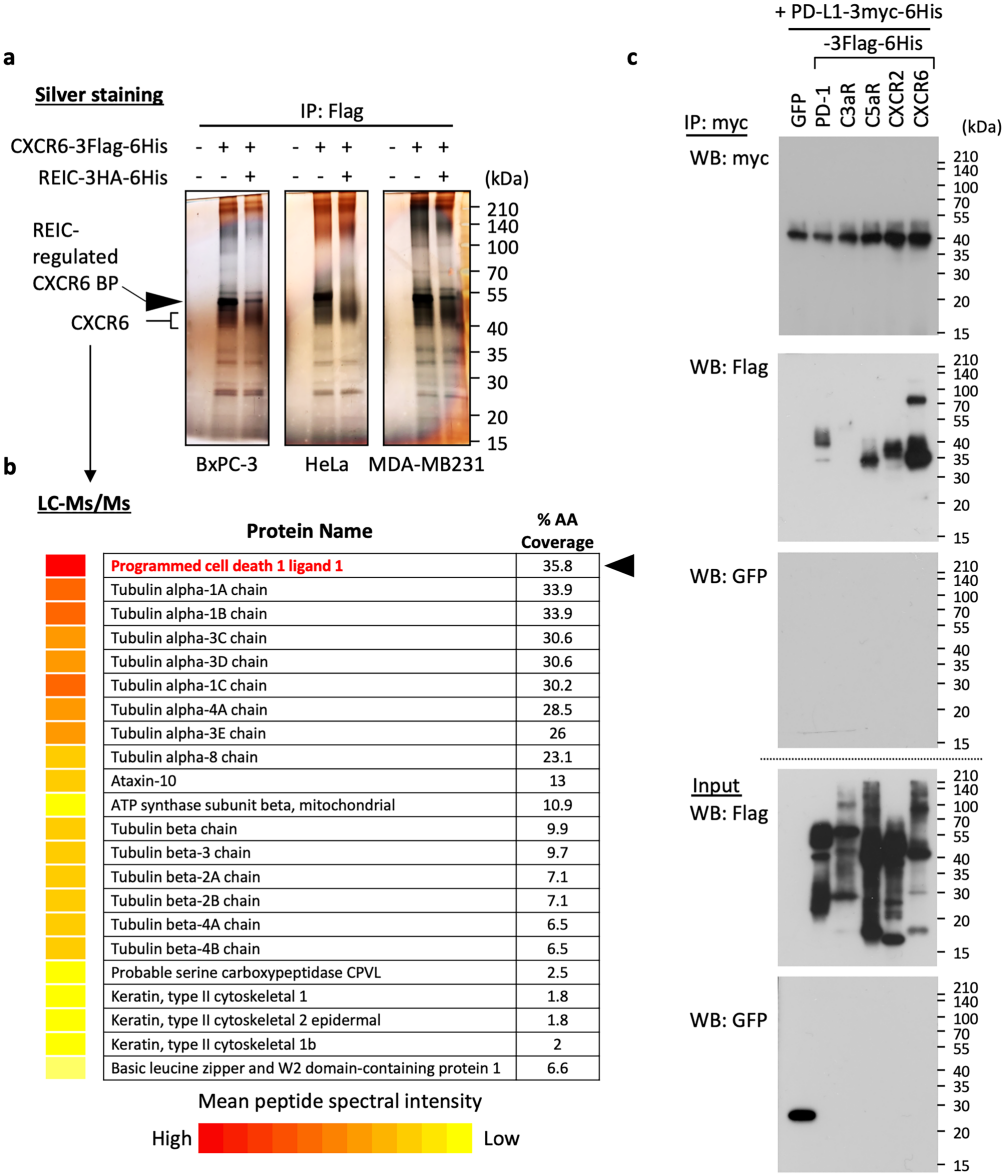
Identification of protein(s) that interact with CXCR6 under the regulation of REIC/Dkk-3. **a** Individual cancer cell lines (BxPC-3, HeLa, and MDA-MB-231) were transfected with the expression plasmids (CXCR6-3Flag-6His and REIC/Dkk-3-3HA-6His) in the indicated combinations. After 36 h, these cells were lysed and pull-downed for the expressed foreign CXCR6, and the precipitated specimens were subjected to SDS-PAGE and then silver staining. **b** A band for a protein that bound with CXCR6 and whose binding was diminished by the presence of REIC/Dkk-3 was subjected to a liquid chromatography-tandem mass spectrometry (LC–MS/MS) analysis; the results are displayed as a modified list. **c** Co-transfection of PD-L1-3myc-6His with individual membrane proteins (PD-1, C3aR, C5aR, CXCR2, CXCR6) tagged with 3Flag-6His or GFP was performed in HEK293T cells. 24 h after the transfections, cell extracts were prepared and then immunoprecipitated using anti-myc tag beads for the expressed PD-L1, and a 4-μl volume of each specimen was analyzed by western blotting

Because the expression of CXCR6 was consistently higher than those of C5aR and CXCR2 in a variety of cancers (Suppl. Fig. S1), we decided to overexpress CXCR6 with or without REIC/Dkk-3 in different types of cancer cells (pancreatic cancer BxPC-3 cells, cervical cancer HeLa cells, and breast cancer MDA-MB-231 cells) (Fig. 2a). The ectopically expressed CXCR6 was immunoprecipitated and the precipitates were subjected to further SDS-PAGE analysis to explore REIC/Dkk-3 regulatable protein(s) that will bind CXCR6, which may provide clues about the role of the interaction between REIC/Dkk-3 and the newly identified receptors. The resulting silver-stained gel focused our attention on one band on the gel (Fig. 2a, indicated by an arrow), which appeared just at the upper side of the expressed CXCR6 band and had significantly lowered intensity due to the co-existence with REIC/Dkk-3 (Fig. 2a). The mass spectrometric analysis of this band unexpectedly resulted in the identification of PD-L1, a protein that suppresses immunity via its interaction with PD-1 (Fig. 2b). The suggested interaction between PD-L1 and CXCR6 was further confirmed by immunoprecipitation in the overexpression system. The experimental setting included two other candidates, C5aR and CXCR2, in addition to CXCR6, two negative controls, green fluorescent protein (GFP) and C3aR, and one positive control, PD-1. The results revealed that all of the candidates exhibited potential to bind with PD-L1 in the manner of PD-1 (Fig. 2c).

### The binding of REIC/Dkk-3 with their interactive membrane protein(s) downregulates PD-L1

**Fig. 3:**
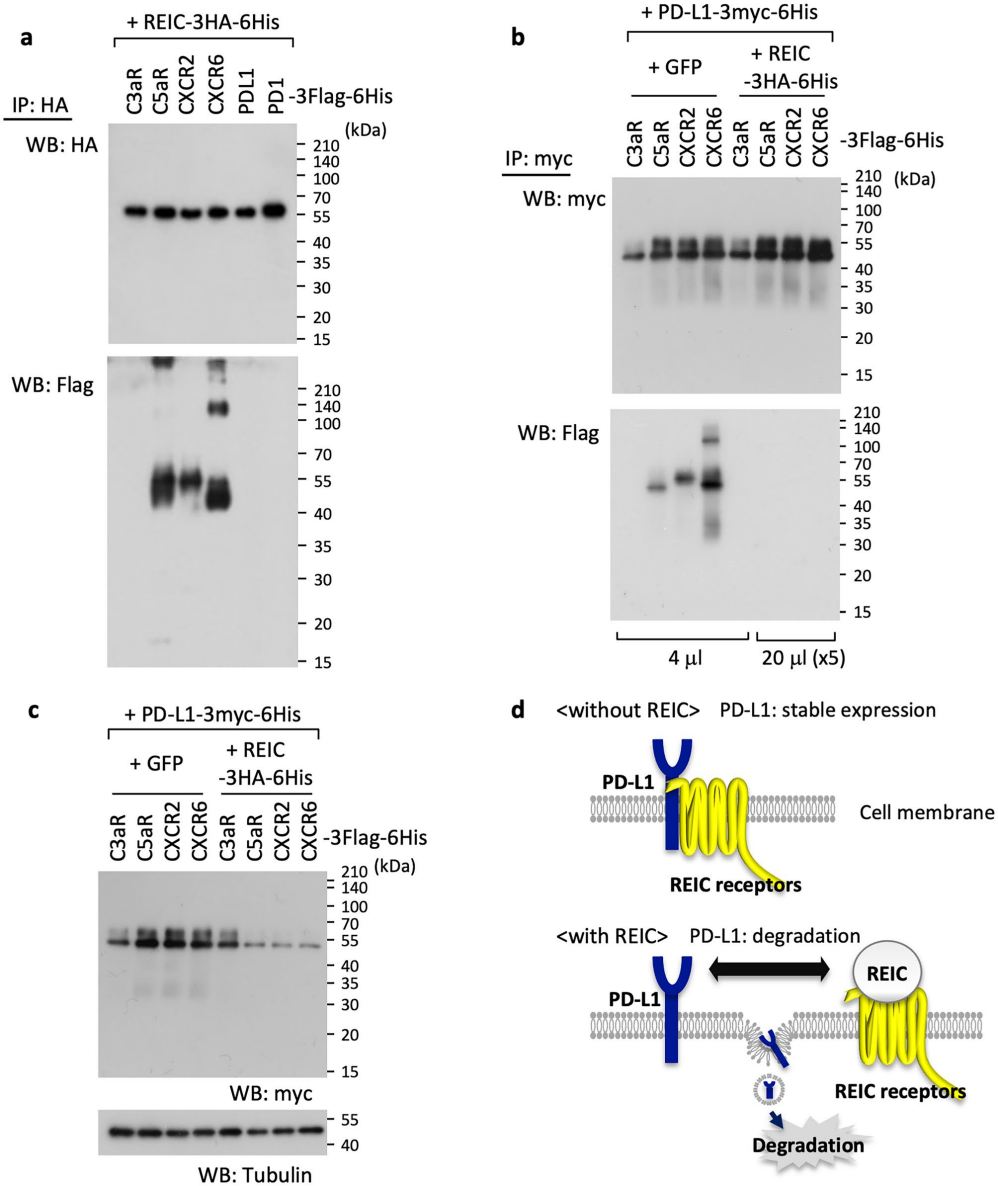
The relationships among REIC/Dkk-3, the identified receptors (C5aR, CXCR2, and CXCR6), and PD-L1. **a** Co-transfection of REIC/Dkk-3-3HA-6His with individual membrane proteins (C3aR, C5aR, CXCR2, CXCR6, PD-L1, and PD1) tagged with 3Flag-6His was performed in HEK293T cells. 24 h after the transfections, cell extracts were prepared and then immunoprecipitated using anti-HA tag beads for the expressed REIC/Dkk-3, and a 4-μl volume of each specimen was analyzed by western blotting. **b, c** HEK293T cells were transfected with the indicated plasmids in different combinations. Cell pellets were collected after 24 h, lysed, immunoprecipitated using anti-Myc tag beads for the expressed PD-L1, and analyzed by western blotting with (**b**) and without (**c**) the immunoprecipitation. For the immunoprecipitated samples (**b**), the sample volume from the REIC-Dkk-3 co-transfection group was 20 μl, which matched a fivefold sample volume from the control GFP co-transfection group. Western blotting was performed using the same volume from the same concentration of the samples adjusted through all preparations (**c**). **d** Schematic representation of the relation among REIC/Dkk-3, REIC receptors, and PD-L1. REIC/Dkk-3 was expected to exert PD-L1 degradation through the binding with the REIC/Dkk-3-target receptors

To confirm some of the results presented in Figs. 1 and 2, we further investigated the above-described bindings with a forced expression system combined with the following immunoprecipitation technique. As shown in Fig. 3a, we confirmed the interaction between REIC/Dkk-3 and all three of the identified receptors C5aR, CXCR2, and CXCR6. An interaction with REIC/Dkk-3 was not observed for the co-expressed PD-L1 or PD-1 or for C3aR, which was used as a negative control. We then used a similar approach to examine the interactions between PD-L1 and the identified receptors in the presence or absence of REIC/Dkk-3. The densitometry results demonstrated that the level of immunoprecipitated foreign PD-L1 was reduced by about fivefold in the presence of the force-expressed REIC/Dkk-3 compared to the values under the GFP-co-overexpression condition (Suppl. Fig. S2, left).

To conduct an immunoprecipitation evaluation using a level of PD-L1 similar to those in the sample preparations, we applied the samples from REIC/Dkk-3 to SDS-PAGE in a higher volume (up to fivefold higher) than used for the GFP group. The approach gave clear bindings of PD-L1 with C5aR, CXCR2, and CXCR6 in the control GFP-co-expressed group, and these were markedly dampened by co-existence with REIC/Dkk-3 (Fig. 3b). The results in Fig. 3b show that, as expected, the expressed PD-L1 levels were significantly lowered by the presence of REIC/Dkk-3 in the sample preparations without immunoprecipitation (Fig. 3c). These results may indicate that (i) REIC-Dkk-3 competitively works to break PD-L1 from the bound receptors, and (ii) the released PD-L1 is immediately moved into a degradation pathway (Fig. 3d).

**Fig. 4:**
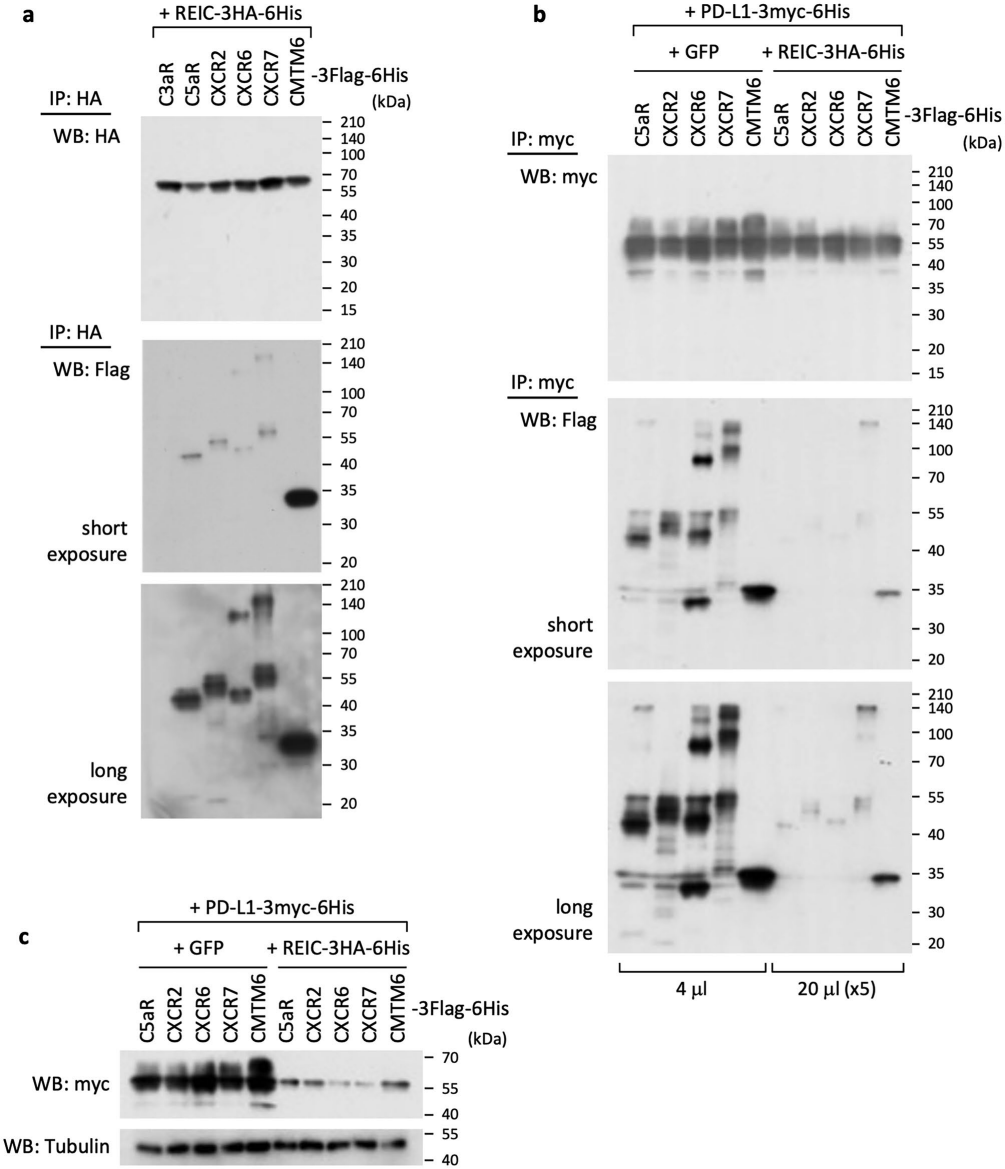
The relationships among REIC/Dkk-3, the potential receptors (CXCR7 and CMTM6), and PD-L1.** a** Co-transfection and a subsequent immunoprecipitation experiment were performed by a method similar to that described in the legend to Fig. 3a, except for the additional use of CXCR7 and CMTM6 expression plasmids. **b, c** Co-transfection, immunoprecipitation, and western blotting were carried out as described in the legend of Fig. 3b and c, except for the use of CXCR7 and CMTM6 expression plasmids

An important regulator of recycling and the plasma membrane expression of PD-L1 has been identified, i.e., CKLF-like MARVEL transmembrane domain-containing protein 6 (CMTM6) [23]. Our identified candidates C5aR, CXCR2, and CXCR6, and probably also CXCR7, may act like CMTM6 toward PD-L1, and REIC/Dkk-3 may modulate the relationship between these receptors and PD-L1. To test this idea, we conducted the same experiments using the method described in the legend to Fig. 3, with the exception of the joining of CXCR7 and CMTM6. The results for CXCR7 and CMRM6 were similar to those observed in the cases of C5aR, CXCR2, and CXCR6; that is, REIC/Dkk-3 (Fig. 4a) and PD-L1 (Fig. 4b) were each able to bind to CXCR7 and to CMTM6 but at different levels.

Our observation of a decrease in the levels of foreign PD-L1 proteins that were immunoprecipitated in the presence of REIC/Dkk-3 (Suppl. Fig. S2, right) prompted us to conduct another evaluation using a higher volume of these proteins in the REIC/Dkk-3-expressing group (Fig. 4b). Similar to the events observed for C5aR, CXCR2, and CXCR6, the bindings with PD-L1 were effectively diminished by the presence of REIC/Dkk-3, and the expressed PD-L1 was highly downregulated in CXCR7 and CMTM6, which we confirmed without immunoprecipitation (Fig. 4c). These results suggest that (i) the stability of PD-L1 is regulated by the bindings with dozens of membrane receptors, including C5aR, CXCR2, CXCR6, and CXCR7 (like CMTM6, whose interactions are negatively modulated by REIC/Dkk-3), and (ii) REIC/Dkk-3 thereby induces the downregulation of PD-L1.

### CMTM-6 is highly expressed in multiple cancer cell species in a consistent manner

**Fig. 5:**
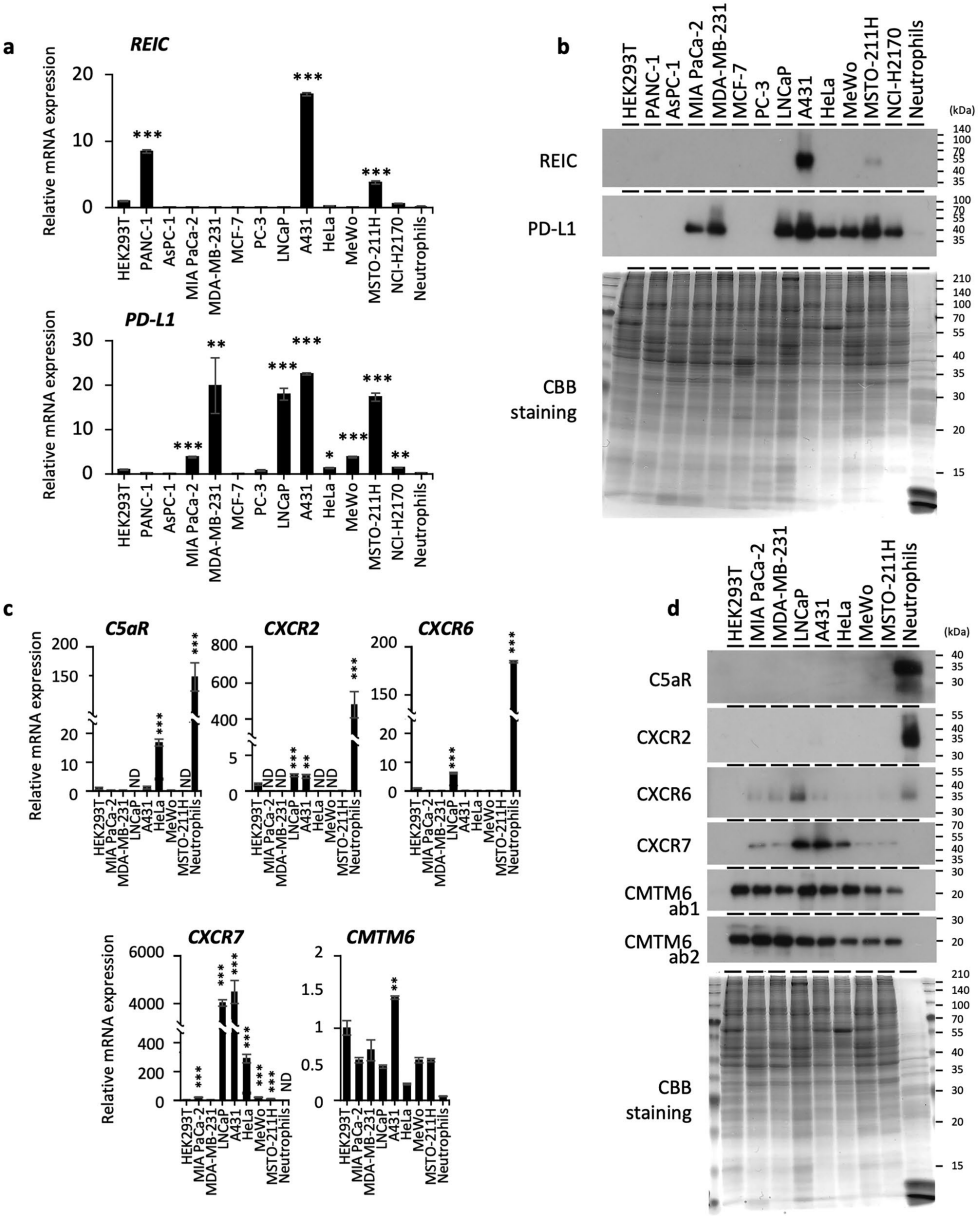
Expression profile of the molecules of interest. **a, b** Total RNAs (**a**) and protein extracts (**b**) prepared from the indicated cell lines (non-cancerous human HEK293T embryonic kidney epithelial cells; human pancreatic cancer PANC-1, AsPC-1, and MIA PaCa-2 cells; human breast cancer MDA-MB-231 and MCF-7 cells; human prostate cancer PC-3 and LNCaP cells; human squamous cancer A431 cells; human cervical cancer HeLa cells; human melanoma MeWo cells; human mesothelioma MSTO-211H cells; human lung cancer NCI-H2170 cells; and human isolated neutrophils from a healthy donor) were analyzed for the expressions of REIC/Dkk-3 and PD-L1. **c, d** The selected PD-L1 highly positive cell lines (MIA PaCa-2 cells; MDA-MB-231cells; LNCaP cells; A431 cells; HeLa cells; MeWo cells; MSTO-211H cells) were further analyzed for their expressions of C5aR, CXCR2, CXCR6, CXCR7, and CMTM6 in comparison to those in HEK293T cells and human isolated neutrophils from a healthy donor by quantitative real-time PCR (**c**) and western blotting (**d**). *ACTB* mRNA was used as a control for the analysis (**c**). Data are mean ± SD. ND, not detected; *p < 0.05, **p < 0.01 by Student’s *t-*test

Because PD-L1 empowers cancer cells to escape from immune surveillance [24], we next investigated which receptor(s) were mainly active in conjunction with PD-L1 in cancer cells. The expression profiling using a quantitative real-time PCR analysis (Fig. 5a) and WB analysis (Fig. 5b) showed that, with the exception of A431 and MSTO-211H cells, none of the PD-L1-positive cells expressed REIC/Dkk-3. We then examined the expression of the candidate receptors in the selected PD-L1-positive cells and found that the expression of each receptor differed among the cell types at both the mRNA and protein levels (Fig. 5c and d). Among them, CMTM6 showed relatively high expression in a consistent manner among all the cancer cell lines examined. These results suggest that (i) CMTM6 is a mainstay of PD-L1 on the surface of cancer cells, and (ii) the other receptors also function cooperatively with CMTM6 to stabilize PD-L1 according to their expression status.

### REIC/Dkk-3 shortens PD-L1 half-life

**Fig. 6:**
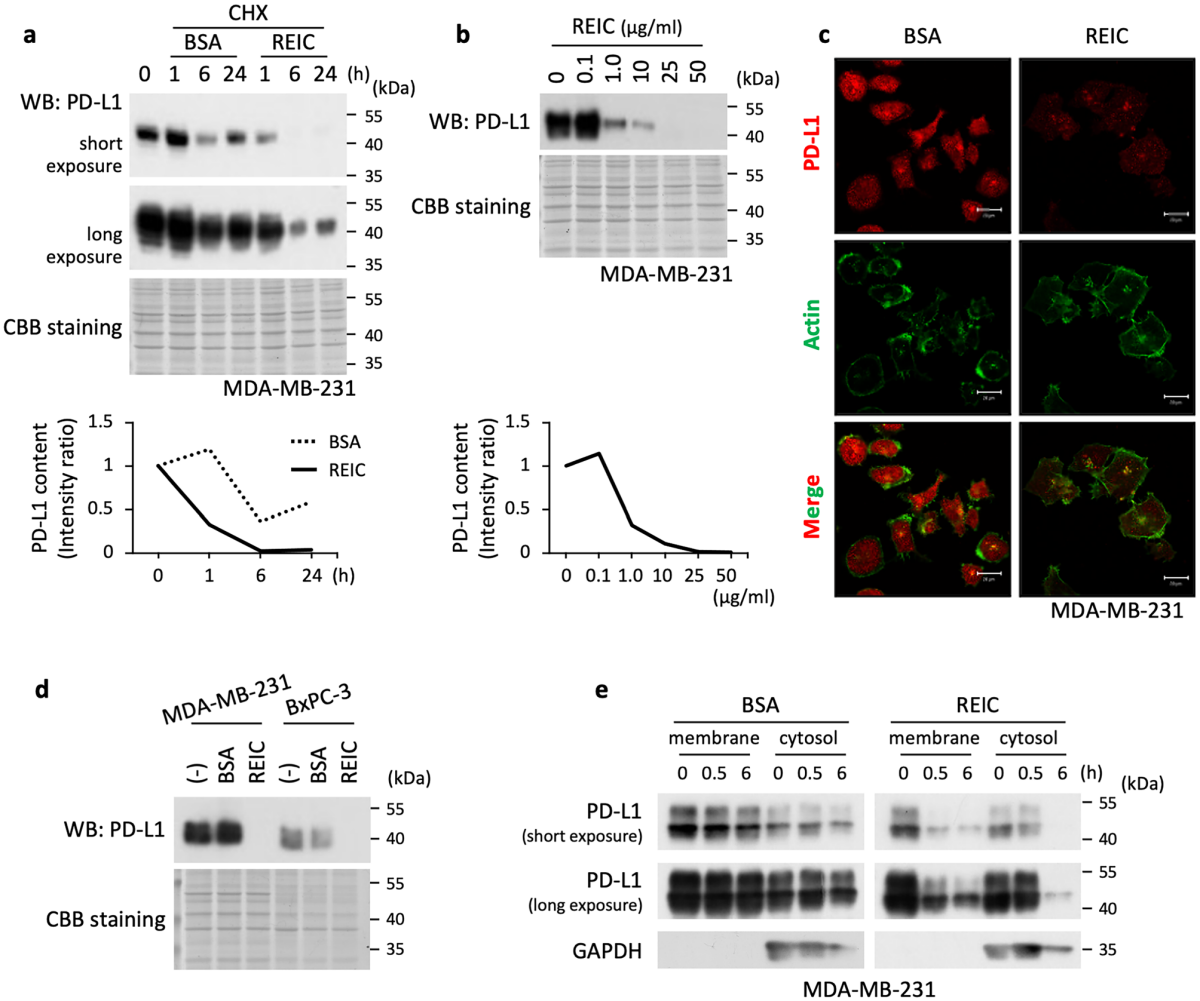
Effects of REIC/Dkk-3 on the half-life of PD-L1 in MDA-MB-231 cells. **a** MDA-MB-231 cells were simultaneously treated with cycloheximide at a concentration of 10 μg/ml and REIC/Dkk-3 recombinant protein at a concentration of 50 μg/ml. After the indicated time intervals, the cells were collected and their intrinsic PD-L1 protein levels were measured (*top*). The results are displayed by declining curves after the normalization of the PD-L1 bands and a subsequent adjustment of the starting points to 0 h and 1.0 as standards (*bottom*). **b** MDA-MB-231 cells were treated together with cycloheximide (10 μg/ml) and the indicated doses of REIC/Dkk-3 recombinant protein (0.1, 1.0, 10, 25, and 50 μg/ml) for 6 h. The western blot of PD-L1 and its quantitated curve are displayed in a manner similar to that described in the legend of Fig. 6a. **c** PD-L1 protein was visualized in MDA-MB-231 cells by immunocytochemistry in which the cells were treated together with cycloheximide (10 μg/ml) and either REIC/Dkk-3 (50 μg/ml) or control BSA (50 μg/ml) for 6 h. Bars: 20 μm. **d** In addition to MDA-MB-231 cells, the cell line BxPC3 was simultaneously treated with cycloheximide (10 μg/ml) and either REIC/Dkk-3 (50 μg/ml) or control BSA (50 μg/ml) for 6 h, and then the treated cells were collected and their intrinsic PD-L1 protein levels were analyzed. **e** MDA-MB-231 cells were treated with cycloheximide (10 μg/ml) and either REIC/Dkk-3 (50 μg/ml) or control BSA (50 μg/ml). After the indicated time intervals, the cells were collected and separated into membrane and cytosol fractions, and the PD-L1 protein levels were analyzed

Our observation that both PD-L1 and CMTM6 were highly expressed in the breast cancer triple-negative MDA-MB-231 cells (Fig. 5), a cell line widely used in PD-L1 studies, led us to use these cells to examine the half-life of PD-L1 after stimulation of the cells with REIC/Dkk-3. As shown in Fig. 6a, the time-course evaluation of the intrinsic PD-L1 protein showed that (i) the protein level was reduced faster in the REIC/Dkk-3-treated cells compared to the control BSA-treated cells, and (ii) the 6-h treatment with REIC/Dkk-3 is required for a high level of reduction. We also observed that the REIC/Dkk-3-induced PD-L1 downregulation started at a concentration of 1.0 μg/ml (Fig. 6b). The reduction of PD-L1 was also confirmed by immunocytochemical analysis of the REIC/Dkk-3 (50 μg/ml, 6 h)-treated MDA-MB-231 cells (Fig. 6c). The REIC/Dkk-3-induced reduction of PD-L1 was not limited to MDA-MB-231 cells; a similar reduction of PD-L1 was observed in pancreatic cancer BxPC-3 cells treated with REIC/Dkk-3 (50 μg/ml, 6 h) (Fig. 6d).

Burr et al. demonstrated that CMTM6 binds with PD-L1 and thereby keeps PD-L1 on the cell surface, and the decreased level of CMTM6 induces accelerated endocytosis of PD-L1 routing for lysosomal degradation [23]. In light of our finding that REIC/Dkk-3 interferes with the binding between CMTM6 and PD-L1, we speculated that REIC/Dkk-3 can lead to PD-L1 endocytosis, which is associated with a faster degradation of PD-L1, similar to the reduction of CMTM6. To examine this, we studied the time course of the PD-L1 status in the two fractionated compartments (membrane and cytosol) after stimulation with REIC/Dkk-3. This approach revealed a much faster transposition of membrane-PD-L1 to the cytosol in the REIC/Dkk-3-treated cells compared to that in the BSA-treated cells at 0.5 h; in addition, the cytosolic PD-L1 following REIC/Dkk-3 treatment became very faint in band intensity at 6 h, which was quite distinct from that in the BSA-treated cells (Fig. 6e). These results suggest that REIC/Dkk-3 competitively binds with CMTM6 to PD-L1, which induces a release of PD-L1 and an immediate endocytosis-mediated degradation of PD-L1.

### REIC/Dkk-3 effectively stalls breast cancer growth in vivo

**Fig. 7:**
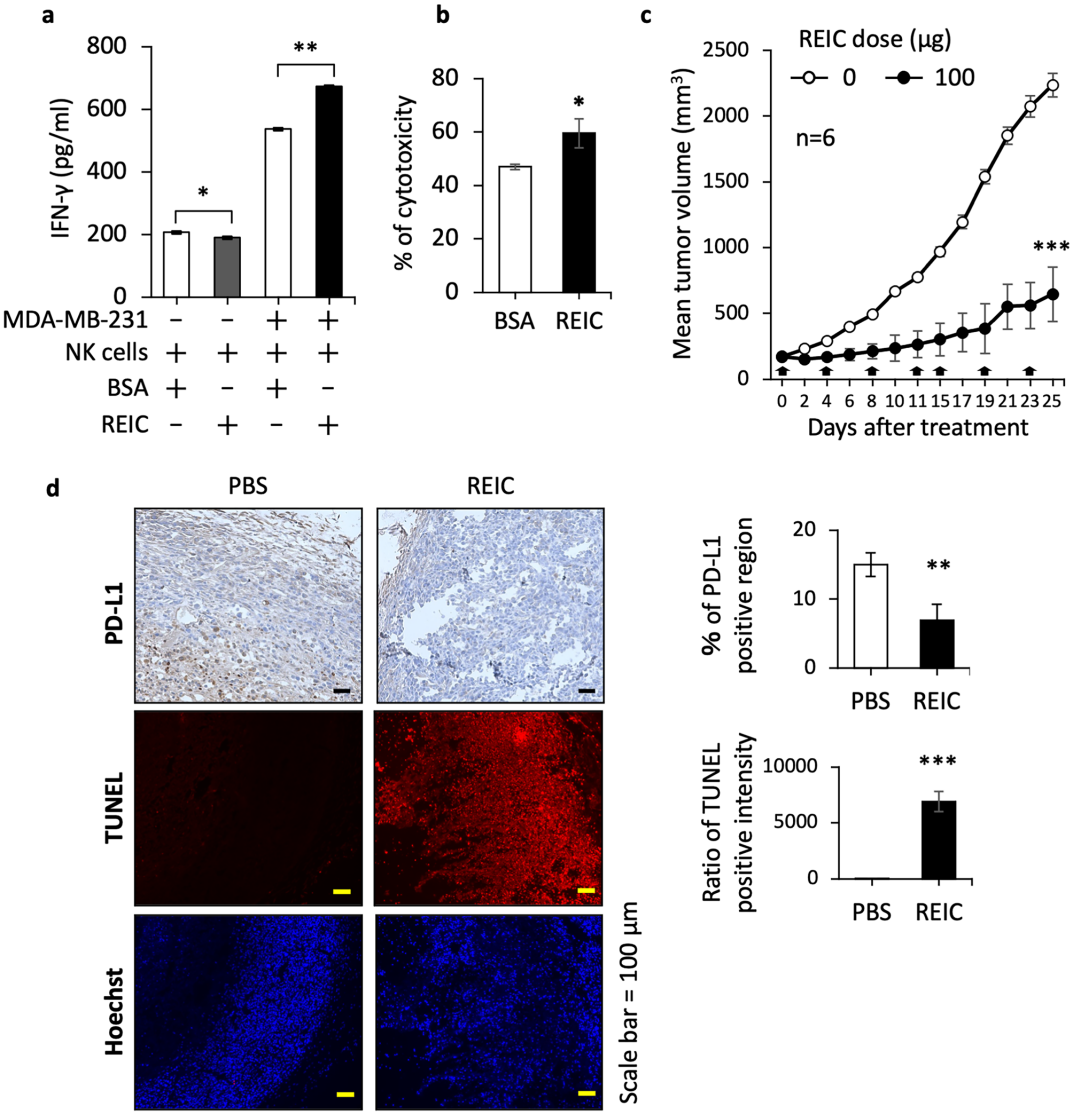
Tumor-suppressing effect of REIC/Dkk-3 in vivo. **a** IFN-γ in culture media was measured at 24 h after the combining of NK cells (5 × 10^4^ cells) with MDA-MB-231 cells (1 × 10^4^ cells). Before the mixed culture of these cells was conducted, NK cells and MDA-MB-231 cells were discretely treated with either REIC/Dkk-3 (50 μg/ml) or control BSA (50 μg/ml) for 24 h. **b** Continuing the experiment described in panel **a**, an NK-cell-mediated cytotoxic assay was performed for MDA-MB-231 cells. The isolated NK cells were pretreated with either REIC/Dkk-3 (50 μg/ml) or control BSA (50 μg/ml) for 24 h, and the treated NK cells (5 × 10^4^ cells) were mixed with MDA-MB-231 cells (1 × 10^4^ cells) for 4 h. **c** The sizes of MDA-MB-231-derived tumors were measured on the indicated days. REIC/Dkk-3 recombinant protein (100 μg) was administered by way of a tail vein starting on day 0 (the tumor volumes reached ~ 150 mm^3^) and then every 4 days until the monitoring was discontinued. **d** On day 16, the resected tumors were prepared for a determination of the intratumor conditions. The status of PD-L1 in the cancer cells was evaluated by staining with an anti-PD-L1 antibody (top). The appearance of apoptotic cells was also evaluated by the staining procedure using the TUNEL method combined with Hoechst-based nuclear staining (bottom). Bars: 50 μm. The stained images at the left were all quantified as shown on the right. Data are mean ± SD. *p < 0.05, **p < 0.01 by Student’s *t-*test

Finally, we examined whether the REIC/Dkk-3-mediated PD-L1 regulation reflects an anticancer immune system. Because the REIC/Dkk-3-mediated mechanism that we observed was validated in MDA-MB-231 cells in vitro (Fig. 6), we subcutaneously engrafted MDA-MB-231 cells in nude mice. As shown in Supplementary Fig. S4a, the intravenous administration of REIC/Dkk-3 recombinant protein significantly worked to stall the engrafted tumor outgrowth in a dose-dependent manner. The cancer PD-L1 is able to dampen not only T cells but also NK cells because these cells consistently express PD-1 [25, 26], and we therefore examined the effect of REIC/Dkk-3 on NK cells in vitro. In this experimental system, we observed that the REIC/Dkk-3-treated MDA-MB-231 cells activate NK cells, as indicated by the increase in IFN-γ (Fig. 7a), and are more fragile to NK cells than the control BSA-treated cancer cells (Fig. 7b). On the other hand, REIC/Dkk-3 had no direct effect on the IFN-γ induction in NK cells (Fig. 7a).

To further examine the NK cell infiltration and cancer cell status in the tumors, we performed an in vivo experiment similar to that shown in Supplementary Fig. S4a, but using a single dose of REIC/Dkk-3 at the highest concentration (100 μg). The tumor suppression occurred right after the treatment of the mice with REIC/Dkk-3 recombinant protein, and the preventive effect continued for 16 days (Suppl. Fig. S4b). The result was confirmed by two additional independent experiments, which were similar to those described above except for the increased number of mice used and the more extended period for the monitoring of tumor outgrowth (Fig. 7c and Suppl. Fig. S4c). Interestingly, microscopic examination of the insides of the REIC/Dkk-3-treated tumors revealed a tissue composition that was consistent with a significant decrease in the number of PD-L1-positive cancer cells (top panel) and an elevated number of apoptotic cancer cells (bottom panel) (Fig. 7d). These results suggest that our clarified novel mechanism of the downregulation of PD-L1 in cancer cells by REIC/Dkk-3 acts in vivo as an anticancer immune system which contributes in part to tumor suppression.

## Discussion

Our previous findings indicate that REIC/Dkk-3 acts as a very strong tumor suppressor in multiple types of cancer. When we force-overexpressed REIC/Dkk-3 in cancer cells by using an adenovirus vector that carries the *REIC/DKK3* gene (Ad-REIC), an effective suppression of cancer outgrowth was observed in vitro as well as in vivo through not only a direct effect that leads to the selective apoptosis of cancer cells but also an indirect effect relevant to immune activation [4–13, 26–28]. The direct effect is caused by REIC/Dkk-3-mediated endoplasmic reticulum (ER) stress via an apoptosis signal-regulating kinase 1 (ASK1)-c-jun N terminal kinase (JNK) pathway in the infected cancer cells [4]. The indirect effect is exerted in at least two ways: one involves the behavior of the mis-infected normal cells with Ad-REIC, which leads to the release of IL-7, a cytokine that activates T cells and NK cells, without apoptosis via an ASK1-p38 pathway; the other involves the secretion of REIC/Dkk-3 at high levels in the infected tumor environment [14].

The extracellularly abundant REIC/Dkk-3 protein induces not only the differentiation of dendritic cells (DCs) from monocytes [2, 3] but also the activation of CD8^+^ T cells and NK cells (our unpublished data). The Ad-REIC-based approach thus greatly contributes to our understanding of anticancer immunity; however, the question of how the extracellular REIC/Dkk-3 protein is involved in anticancer immunity at the molecular level remains. Clues to solving this important issue are likely to depend on the identification of REIC/Dkk-3 receptor(s). Our present experiments identified multiple chemokine receptors (C5aR, CXCR2, CXCR6, and CMTM6) other than the previously reported CXCR7 as novel binding partners of REIC/Dkk-3 on the cell surface, and we observed that the binding interactions did not involve agonistic or antagonistic effects on the receptors under our experimental conditions, but did have a significant role in PD-L1 regulation. Our results revealed that extracellular REIC/Dkk-3 protein makes PD-L1 break away from the chemokine receptors that sequester PD-L1 in the cytoplasmic compartment, leading to an accelerated degradation of PD-L1; cancer immune evasion is thereby dampened. This insight provides further evidence suggesting the possibility of plural anticancer functions of REIC/Dkk-3.

Among the identified receptors, CMTM6 may be especially important in cancer cells since it was expressed at significant levels in cancer cells in a manner consistent with the expression of other receptors (Fig. 5). Other research groups have been examining the interaction of CMTM6 with PD-L1 in cancer cells; they revealed that CMTM6 associates with PD-L1 on the cell surface, reduces its ubiquitination, increases the half-life of PD-L1 [29], and acts to exacerbate anticancer immunity [23]. The interaction described herein is thus not new, but the present data strongly support our speculation that PD-L1 stability is maintained by the identified receptors, including CMTM6, and that the variations in the interactions of these receptors are likely to be dependent on the cell types because of the different expression levels of the receptors (Fig. 5). The new insight of our present study is that the extracellular REIC/Dkk-3 breaks down the interactions. Thus, REIC/Dkk-3 contributes to the induction of cancer vulnerability against immune cells. However, the general principle of the competitive binding of REIC/Dkk-3 and PD-L1 to these multiple receptors in a consistent manner is still a closed book since no appreciable domain that is commonly shared by the five receptors was observed (Suppl. Fig. S3). In addition, there was no amino acid-alignment region with high homology among these receptors (data not shown). Therefore, more advanced analyses will be needed to determine the common binding principle of these complex interactions, such as investigations based on the protein crystal structures.

In our present in vivo experiments, we faced several technical problems with the use of a syngeneic allograft model of cancer. For example, it was difficult to establish a reliable experimental method to induce cancer-recognizing CD8^+^ T cells in vivo, and we could not establish well-matched mouse cell lines for our experiments because PD-L1 was expressed at a low level or not at all in the cell lines available to us, i.e., breast cancer 4T1 cells [30], melanoma B16-BL6 cells [31], and colorectal Colon-26 cells (our unpublished data). We therefore turned to a nude mouse-based xenograft model using human MDA-MB-231 cells for the REIC/Dkk-3 protein-mediated anticancer evaluation. With this model, we observed marked tumor suppression with a significant downregulation of PD-L1 in cancer cells in the tumors in the REIC/Dkk-3 treated mice (Fig. 7c and d). This effect was due at least in part to an unweakened NK cell function because PD-L1 on the cancer cell side was dampened by REIC/Dkk-3, and we confirmed that PD-1 on NK cells may not be strongly affected by the cancer PD-L1 (Fig. 7b). We confirmed that REIC/Dkk-3 has no direct effect on the NK cell side as part of its anticancer activity (Fig. 7a).

The above-described function of REIC/Dkk-3 may be reflected (at least in part) by the cancer suppression in clinical cancer treatments with Ad-REIC. In the First-In-Human phase I/IIa study at Okayama University Hospital, Kumon et al. reported that the highest dose of Ad-REIC used in this therapeutic context surprisingly achieved a high cure rate in a group of patients with high-risk prostate cancer, with no severe side effects [15–17]. Of note, Kumon et al. found that in the treated tumor tissue, there were too many apoptotic cancer cells along with highly increased numbers of infiltrating CD8^+^ T cells and dendritic cells, implying a non-stalled effective attack on the neighboring cancer cells by CD8^+^ T cells.

In contrast to the potential benefits of REIC/Dkk-3, we need to consider the shortcomings of the use of this protein for cancer treatment. It might be useful to tailor Ad-REIC treatment for specific purposes. Any cancer milieu is composed of many types of cells — not only cancer cells but also normal cells, including inflammatory immune cells, fibroblasts, endothelial cells, and more — whose cell–cell cross-talk plays a crucial role in cancer progression [32]. We suspect that the REIC/Dkk-3 protein exerts diverse functions that depend on the cell type. Untergasser et al. reported that REIC/Dkk-3 is highly expressed in and secreted from cancer-associated endothelial cells, which in turn stimulates endothelial cells in an autocrine manner, leading to the angiogenesis that is important for the supply of nutrition to cancer cells for their outgrowth [33]. These events require the ALK1 receptor, which induces an active production of vascular endothelial growth factor (VEGF) through the enhanced phosphorylation of Smad1/5/8 upon REIC/Dkk-3 binding to the receptor [34]. The cancer stroma is filled by matrix, with approx. 90% being occupied by fibroblasts called cancer-associated fibroblasts (CAFs), which actively contribute to cancer aggressive behaviors [35].

REIC/Dkk-3 is also actively secreted from CAFs, and the secreted protein facilitates the activation of CAFs [36]. The REIC/Dkk-3 receptor on CAFs is the cell surface Kremen, a negative regulator of canonical Wnt signaling. The binding of REIC/Dkk-3 with Kremen enhances the cell-surface levels of low-density lipoprotein receptor-related protein 6 (LRP6), and thereby the LRP6-mediated signal is upregulated upon Wnt binding, eventually leading to enhanced CAF outgrowth and the mal-production of cancer preferential matrices. REIC/Dkk-3 thus has diverse roles in different cell types in different cancer milieus that lead to cancer suppression or cancer progression, or both at the same time depending on the plural in situ contexts, because of the presence of multiple receptors to the extracellular REIC/Dkk-3. We have observed that the tumor-suppressing effect of REIC/Dkk-3 protein in mouse models is far higher in MDA-MB-231 cells (Fig. 7c, d) than that in Colon-26 cells (our unpublished data). This may be due to the abovementioned diverse roles of REIC/Dkk-3 protein; investigating this possibility is a goal of our ongoing research.

In conclusion, our present study proposes a novel function of the REIC/Dkk-3 protein: a negative regulation of PD-L1 on the cancer cell surface as a tumor-suppressive effect that acts against PD-L1-mediated cancer immune evasion. Our findings help explain a portion of the significant Ad-REIC-mediated effect of cancer suppression. A problem remains, however: the REIC/Dkk-3 protein promotes cancer in a few cases. Further research is necessary to overcome this, and a key task is to first comprehend the individual interactions of the REIC/Dkk-3 protein with the multiple receptors at the molecular level. A general understanding of these interactions will contribute to the development of an innovative REIC/Dkk-3 protein of the desired specificity. Such amelioration engineering of REIC/Dkk-3 will help improve the future applications of Ad-REIC cancer gene therapy.

## Supplementary Information

Below is the link to the electronic supplementary material.

Supplementary file1 (TIF 61.0 MB)

Supplementary file1 (TIF 61.0 MB)

Supplementary file1 (TIF 61.0 MB)

Supplementary file1 (TIF 61.0 MB)

## Data Availability

The raw data supporting the conclusions of this article will be made available by the authors, without undue reservation.

## Abbreviations

Ad: Adenovirus
ASK1: Apoptosis signal-regulating kinase 1
CAF: Cancer-associated fibroblast
C3aR: Complement C3a receptor 1
C5aR: Complement C5a receptor 1
CMTM6: CKLF-like MARVEL transmembrane domain-containing protein 6
CXCR: C-X-C chemokine receptor type
DKK3: Dickkopf WNT signaling pathway inhibitor 3
DP: Prostaglandin D2 receptor
ELISA: Enzyme-linked immunosorbent assay
EP: Prostaglandin E receptor
ERK: Extracellular signal-regulated protein kinase
FPR: Formyl peptide receptor 1
IL: Interleukin
IRF1: Interferon regulatory factor 1
JNK/SAPK: C-Jun N-terminal kinase/stress-activated protein kinase
LC–MS/MS: Liquid chromatography-tandem mass spectrometry
LPAR: Lysophosphatidic acid receptor
LRP6: Low-density lipoprotein receptor-related protein 6
LTR: Cysteinyl leukotriene receptor
PCR: Polymerase chain reaction
PD-L1: Programmed cell death 1 (PD-1) ligand 1
REIC: Reduced expression in immortalized cells
SDS-PAGE: Sodium dodecyl sulfate–polyacrylamide gel electrophoresis
STAT1: Signal transducers and activators of transcription 1
TAM: Tumor-associated macrophage
TUNEL: Terminal deoxynucleotidyl transferase (TdT)-mediated dUTP nick-end labeling
VEGF: Vascular endothelial growth factor
