# Supplementary figures and images for: Novel extracellular role of REIC/Dkk-3 protein in PD-L1 regulation in cancer cells
Source: J Mol Med (Berl). 2023 Mar 4;101(4):431–47. doi: 10.1007/s00109-023-02292-w (PMC10090029; doi:10.1007/s00109-023-02292-w)

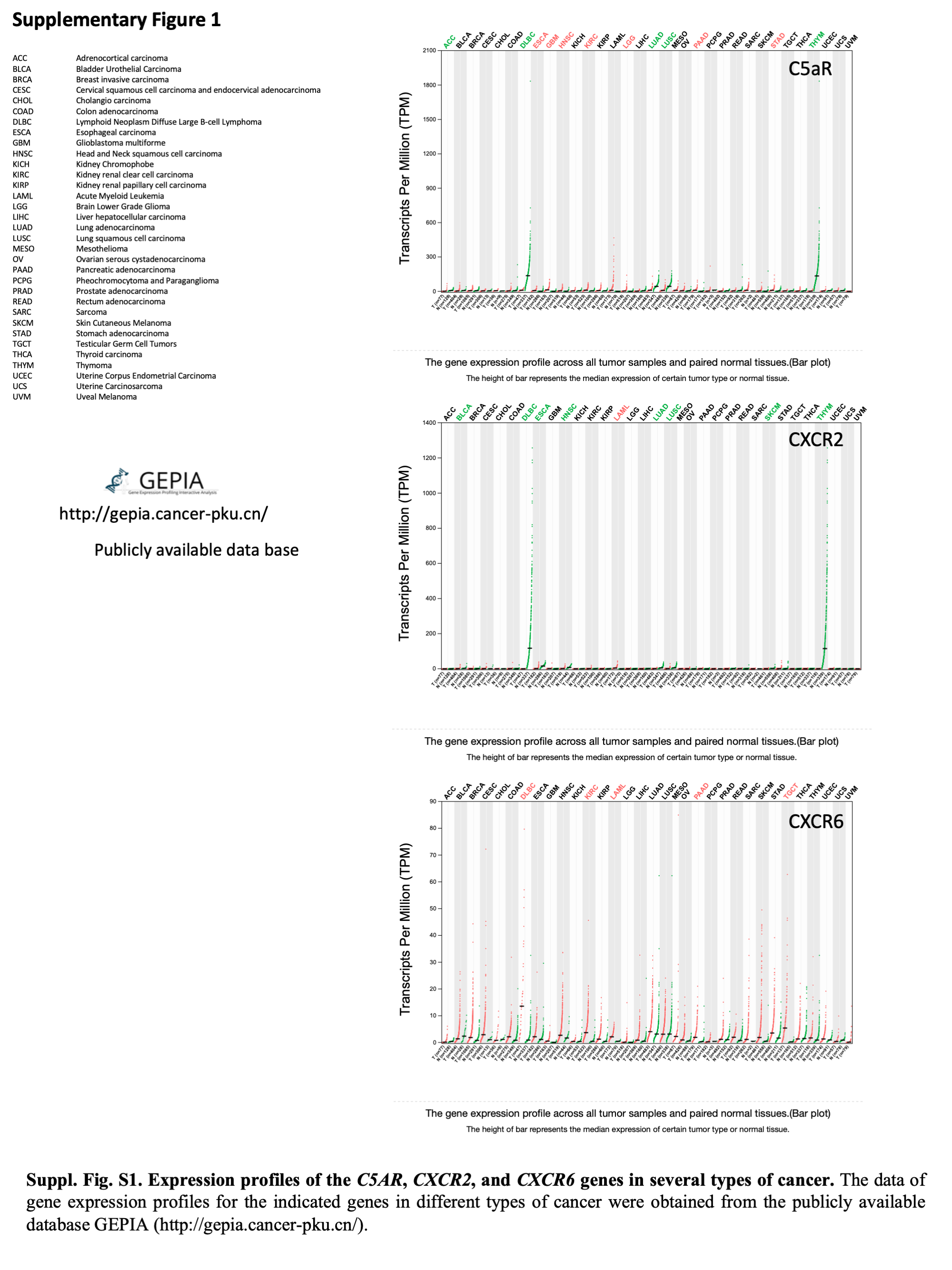

Supplement: Supplementary file 1 — Supplementary file1 (TIF 61.0 MB) [file 109_2023_2292_MOESM1_ESM.tif]

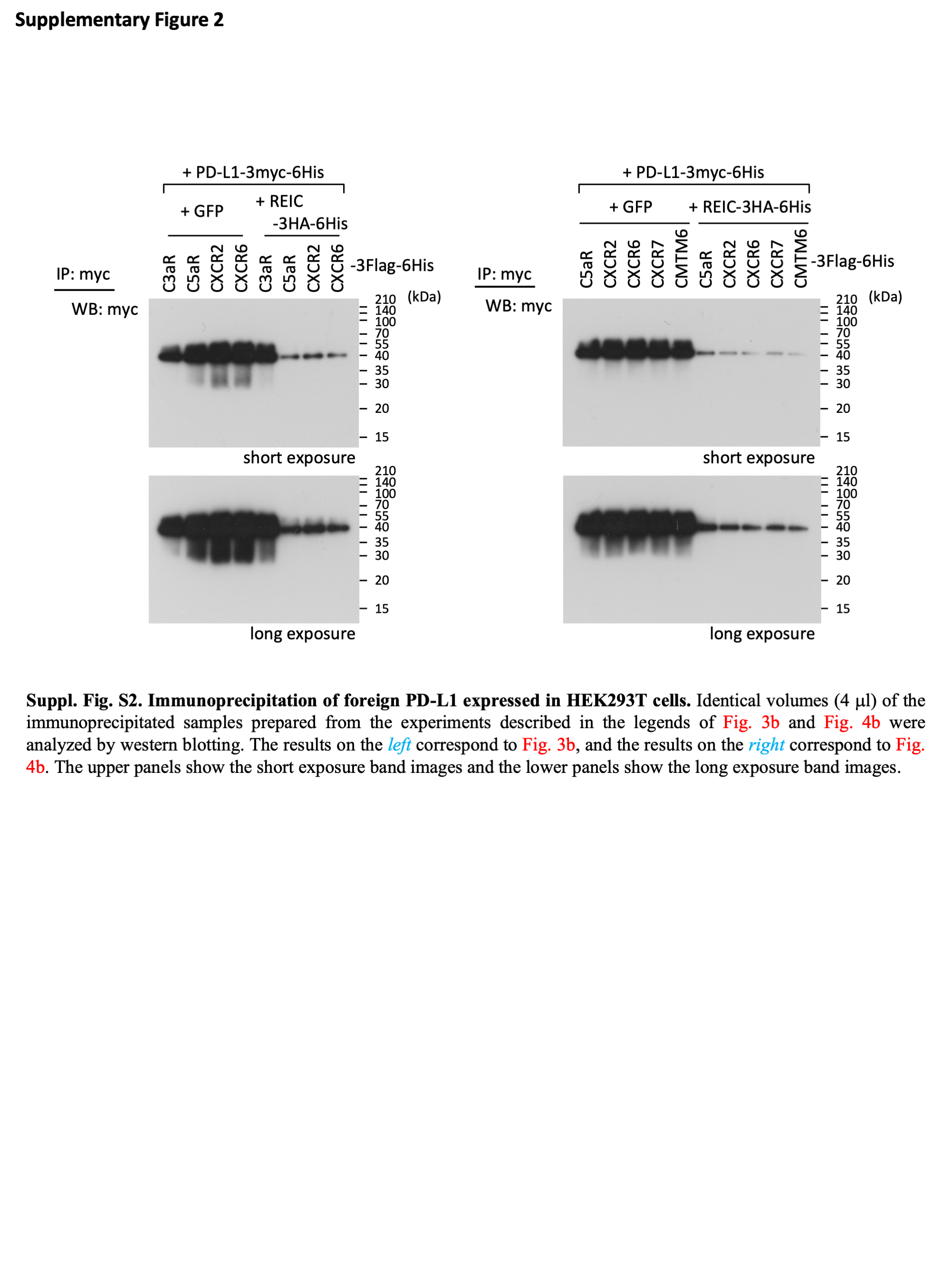

Supplement: Supplementary file 2 — Supplementary file1 (TIF 61.0 MB) [file 109_2023_2292_MOESM2_ESM.tif]

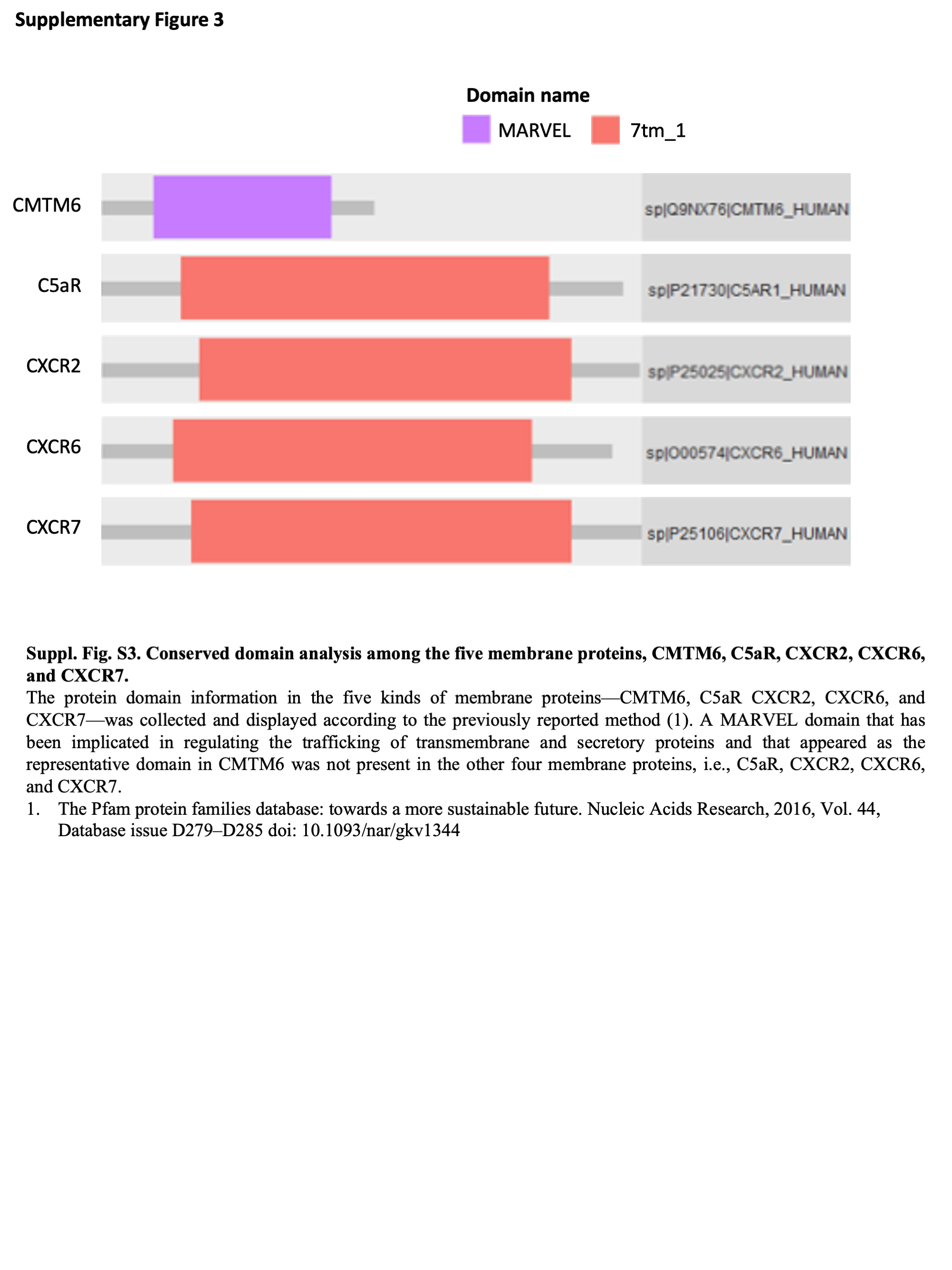

Supplement: Supplementary file 3 — Supplementary file1 (TIF 61.0 MB) [file 109_2023_2292_MOESM3_ESM.tif]

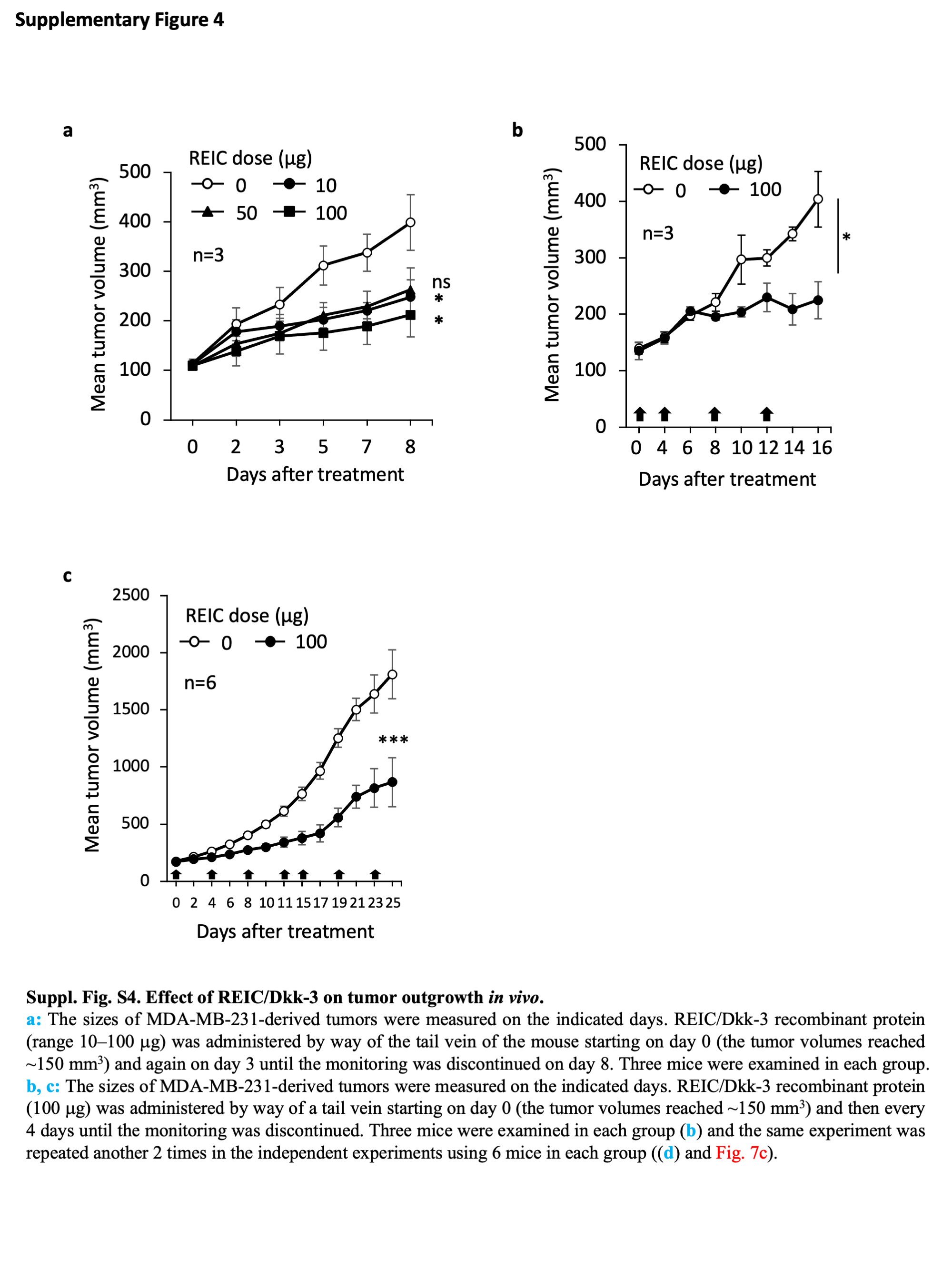

Supplement: Supplementary file 4 — Supplementary file1 (TIF 61.0 MB) [file 109_2023_2292_MOESM4_ESM.tif]
